# Critical Functions of Region 1-67 and Helix XIII in Retaining the Active Structure of NhaD Antiporter in *Halomonas* sp. Y2

**DOI:** 10.3389/fmicb.2018.00831

**Published:** 2018-05-02

**Authors:** Zhou Yang, Yiwei Meng, Qi Zhao, Bin Cheng, Ping Xu, Chunyu Yang

**Affiliations:** State Key Laboratory of Microbial Technology, Microbial Technology Institute, Shandong University, Jinan, China

**Keywords:** NhaD antiporter, *in vivo* activity, fusion protein, TM XIII, pH activation

## Abstract

NhaD-type antiporters are mainly distributed in various *Proteobacteria*, especially in marine microorganisms and human pathogens. This distribution as well as the pathogenic properties of these strains suggest that these antiporters contribute to the regulation of high osmoregulation and are potential drug targets. Two NhaD homologs, NhaD1 and NhaD2, from the halotolerant and alkaliphilic *Halomonas* sp. Y2 exhibits similar, high *in vitro* activity, but remarkably different *in vivo* functions. To search for critical domains or residues involved in these differences of physiological functions, various chimeras composed of NhaD1 and NhaD2 segments were generated. Two regions at residues 1–67 and 464–492 were found to be responsible for the robust *in vivo* function of NhaD2, and region 464–492 is also crucial to the pH response of the antiporter. In particular, the completely abolished activity of KNabc/N463r, highly recovered activity while very weakly recovered ion resistance of the KNabc/N463r-C7 chimera, suggested that transmembrane helix (TM) XIII is crucial for the robust ion resistance of NhaD2. Using site-directed mutagenesis, seven hydrophobic residues in TM XIII were identified as key residues for the ion translocation of NhaD2. Compared with the fluorescence resonance energy transfer (FRET) profile in the wild-type NhaD2, the reduced FRET efficiency of N463r chimeras provided solid evidence for conformational changes in the N463r fusion protein and consequently verified the structural functions of TM XIII in the pH activation and physiological functions of NhaD2.

## Introduction

Sodium proton (Na^+^/H^+^) antiporters are secondary membrane protein transporters present in taxa belonging to all kingdoms. Over the last few decades, many putative Na^+^/H^+^ antiporters have been identified in a wide range of taxa and their critical functions have been characterized, e.g., in sodium, pH, and cell volume homeostasis; furthermore, they have been identified as potential drug targets in humans (Padan et al., 2001; Padan and Landau, 2016). Among these antiporters, NhaA from *Escherichia coli* (Ec-NhaA) has been extensively investigated with respect its structural properties, ion translocation activity, and pH regulatory effects (Maes et al., 2012). Since the crystal structure of downregulated Ec-NhaA was determined at pH 4.0, many biochemical and physiological studies have been performed and the pH-dependent features of Ec-NhaA have been elucidated (Hunte et al., 2005; Krulwich et al., 2011). In particular, the pH- and Na^+^-induced conformational changes and working model of Ec-NhaA have been monitored using various techniques, and these studies have provided solid evidence that environmental stress induces conformational changes of Ec-NhaA (Kozachkov et al., 2007; Tzubery et al., 2008; Appel et al., 2009; Herz et al., 2010; Mager et al., 2011).

Based on the TCDB^1^ and PubMed databases, NhaD-type homologs have been exclusively found in the cell membranes of marine/haloalkaliphilic γ-*proteobacteria* and pathogenic strains, including *Vibrio cholera, Chlamydia trachomatis, Prevotella bergensis*, and *Campylobacter showae*. This distribution suggests a special mechanism for saline (alkaline) habitat adaptation and implies their potential application as drug targets (Kurz et al., 2006). However, only limited information of these antiporters are available from halotolerant or alkaliphilic species. Transport activity analyses of those antiporters from *Alkalimonas amylolytica*, *Halomonas*, and pathogenic *Vibrio* species have suggested that they are highly pH-dependent. In addition, these NhaD antiporters exhibited different pH profiles for Na^+^ and Li^+^ transport (Nozaki et al., 1998; Dzioba et al., 2002; Liu et al., 2005; Zhang et al., 2014), consistent with our previous findings for NhaD antiporters of the strain *Halomonas* sp. Y2 (Cui et al., 2016).

In the halotolerant and alkaliphilic *Halomonas* sp. Y2, we found Na^+^/H^+^ antiporters of different types working in concert to adapt to alkaline or saline stresses (Cheng et al., 2016). Among four antiporters investigated, two NhaD homologs (sequence identity, 72%) displayed substantially different physiological functions, i.e., NhaD2 exhibited robust physiological functions in both wild-type *Halomonas* sp. Y2 and antiporter-deficient *E. coli* strain KNabc, whereas very weak *in vivo* activity was detected for NhaD1 (Cui et al., 2016). Previously, we found that the N- and C-termini of these two NhaD antiporters functionally interact and play important roles in expelling ions (Meng et al., 2017), but the critical regions for these *in vivo* roles remain to be elucidated. In this study, to advance our understanding of the molecular mechanism underlying these physiological differences, critical regions and residues were identified by constructing a series of chimeras and mutants.

## Materials and Methods

### Bacterial Strains, Plasmids, and Growth Conditions

The bacterial strains and plasmids used in this study are listed in **Table 1** and Supplementary Table S1. *E. coli* strain DH5α was used for routine cloning and cultivated at 37°C, in the Luria–Bertani (LB) medium containing 1.0% tryptone, 0.5% yeast extract, and 1.0% NaCl. The triple-antiporter deficient strain *E. coli* KNabc (Nozaki et al., 1998) was used as a host for the complementary assay and membrane vesicle preparation. Unless otherwise specified, *E. coli* KNabc transformants were grown aerobically at 37°C in the LBK medium, in which NaCl was replaced by 87 mM KCl and 100 μg ml^-1^ ampicillin was supplemented (Goldberg et al., 1987).

### Chimera and Mutant Construction

**FIGURE 1:**
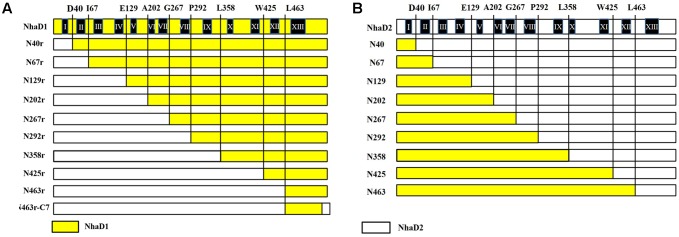
Schematic representation of NhaD1 and NhaD2 chimeras. **(A)** NhaD1 chimeras that replaced N-terminal regions of NhaD1 with corresponding regions of NhaD2. **(B)** NhaD2 chimeras that replaced N-terminal regions of NhaD2 with corresponding regions of NhaD1. Regions from NhaD1 and NhaD2 are colored with yellow and white, respectively.

The amino acid alignment between NhaD1 and NhaD2 was performed by Clustal_X (Thompson et al., 1997). For chimera generation (**Figure 1**), the gene splicing by overlap extension PCR (OE-PCR) was used for fusion fragments construction, by using NhaD1 and NhaD2 as templates, respectively. The primers used for fusion PCR was listed in Supplementary Table S2. These fused fragments were ligated into pEASY-blunt and transformed into *E. coli* DH5α for sequencing. After been confirmed for sequence fidelity, each fusion plasmids were transformed into *E. coli* KNabc for complementary growth and antiport activity measurement.

To recover the complementation ability of N463r, the three C-terminal residues of N463r (SMF) were replaced with seven C-terminal residues of NhaD2 (GSFSVYG), and a fusion protein of N463r-C7 was generated (**Figure 1**). Using the primers listed in Supplementary Table S2 and plasmid pEASYblunt-N463r-C7 as the template, seven mutants in transmembrane helix (TM) XIII were generated by PCR-based technique. Fidelity of all final mutated versions of *nhaD* in pEASY-blunt was verified by DNA sequencing. *E. coli* KNabc recombinants carrying the mutated plasmids were constructed as those of chimeras.

### Growth of Constructed *E. coli* KNabc Variants

Complementary growth of a series of chimeras and mutants, together with KNabc/NhaD1 and KNabc/NhaD2, were tested in LBK medium supplemented with 200, 300 mM NaCl or 50, 100 mM LiCl, respectively. Recombinant carrying empty vector pEASY-blunt in *E. coli* KNabc was used as a negative control. Ampicillin was added to a final concentration of 100 μg ml^-1^. After incubation at 37°C for 24 h, the growth of strains (OD_600_) was measured.

### Preparation of Reversed Membranes and Determination of Transport Activity

The KNabc mutants were cultured in LBK medium (100 μg ml^-1^ ampicillin) at 37°C to a concentration (OD_600_ = 0.6), and then IPTG (final concentration of 0.5 mM) was added for protein induction. Inside-out membrane vesicles from these cultures were prepared and the fluorescence-based activity was measured as previous described (Goldberg et al., 1987; Cheng et al., 2016). Protein concentration was determined by the Bradford protein assay (Bio-Rad). The antiport activity of around 60 μg vesicle was assayed in buffer B (140 mM choline chloride and 5 mM MgCl_2_, 10 mM MES, 10 mM Tris, pH 8.5), by measuring the dequenching of fluorescence upon the subsequent addition of 10 mM of Na^+^ or Li^+^. The percent dequenching was calculated relative to the initial respiration-dependent quench. For the pH profile measurement, assay mixtures containing of 10 mM Tris-MES and 10 mM of Na^+^ (Li^+^) at different pH conditions were used (6.0–9.5). The concentration ranges of the cations tested was 0.1–100 mM at pH 8.5, and the apparent *K*_m_ values were calculated by linear regression of a Lineweaver–Burk plot.

### Complementary Strain Construction in *Halomonas* sp. Y2/Δ*nhaD2*

N463r-C7 fused fragments, as well as its seven mutants were ligated with the shuttle vector pBBR1MCS-5, and transferred into the *nhaD2*-deficient strain of *Halomonas* sp. Y2 using the method we previously described (Cheng et al., 2016). The transformants were selected by LB plates with ampicillin (100 μg ml^-1^) and gentamicin (50 μg ml^-1^) supplementation, and confirmed by PCR and DNA sequencing.

The growth of eight complementary strains upon high alkalinity and salinity were measured in liquid LB-based medium that buffered with 50 mM Tris-HCl (pH 8.0) or 50 mM glycine-NaOH (pH 10.0), respectively. Based on the ion- and alkali-resistance of *Halomonas* sp. Y2, various concentrations of NaCl and LiCl were supplemented. For seed preparation, all cell suspensions were adjusted to 6.0 (OD_600_) with Na^+^-free LB medium and inoculated in a same inoculation volume. After 24 h incubation at 37°C, the cell growth (OD_600_) was determined.

### RNA Preparation and Quantitative Real-Time Reverse Transcription PCR (qRT-PCR) Analysis

The relative qPCR was performed to calculate the expression levels of N463r-C7 and its seven mutated proteins in helix XIII (TM XIII). Strains of KNabc/D1, D2, N463r-C7 and its mutants were cultured in LBK medium at 37°C to late exponential phase. Then, cells were harvested and used for RNA extraction, by using EasyPure RNA kit (Transgen). The cDNA was synthesized by the HiScript Q RT SuperMix for Qpcr (Vazyme Biotech) and used as the templates for qPCR analysis. Quantitative PCR was carried out in a MyiQ2^TM^ iCycler (two-color real-time PCR detection system) (Bio-Rad) with a Real Master mix kit (SYBR Green, Tiangen) according to the manufacturer’s instructions. The primers used in this procedure are listed in Supplementary Table S2 and *16S rRNA* was used as an internal reference for each mutant. Three biological replicates were used for qPCR and at least four repeats for each sample, after which the average threshold cycle (Ct) was calculated for each sample. By using the Ct values of *nhaD2* as a baseline, the relative fold changes in gene expression were calculated by 2^-ΔΔCt^ method. Statistical analysis was performed on 2^-ΔΔCt^ values using a paired Student’s *t*-test.

### Fluorescence Resonance Energy Transfer (FRET) Analysis for the Conformational Change

A previously construct of N39-CFP-C-YFP (designated as D2-CFP-YFP in this study) was also used as a template for generating mutant N463r-CFP-YFP (Meng et al., 2017), in which TM XIII was replaced with the corresponding region of NhaD1, CFP was fused after site 39 and YFP was fused at the C-terminal of N463r. In these two fusion proteins, CFP and YFP were fused after site 39 and C-terminus, respectively. *E. coli* KNabc carrying these plasmids were tested for antiport activities. After activity confirmation, *E. coli* C43 strains carrying these fusion plasmids were grown at 37°C in LB medium to a concentration (OD_600_ = 0.6–0.8) and induced by adding IPTG (final concentration of 0.5 mM), and cultivated at 20°C overnight. Cells were harvested and washed three times with the Tricine-KOH buffer solution (10 mM Tricine and 140 mM KCl, adjusted to pH 8.5 with KOH), and resuspended in the same buffer to an OD_600_ of 1.5. For fluorescence resonance energy transfer (FRET) analysis, a 2-ml cell suspensions of D2-CFP-YFP, N463r-CFP-YFP, or D2-CFP were irradiated at 433 nm to excite CFP, and recorded the fluorescence emission at 450–600 nm on a F-4600 spectrofluorophotometer (Hitachi). In the meanwhile, a 2-ml cell suspension of D2-CFP-YFP was also illuminated at 473 nm and recoded at 450–600 nm.

## Results

To identify the precise regions involved in the physiological differences, a series of chimeras were generated, as shown in **Figure 1**. The N-terminal region of NhaD1 and C-terminal sequence of NhaD2 were combined, or vice versa. As a result, two sets of chimeras from NhaD1 and NhaD2 were both generated.

### *E. coli* KNabc Chimeras of NhaD1

**FIGURE 2:**
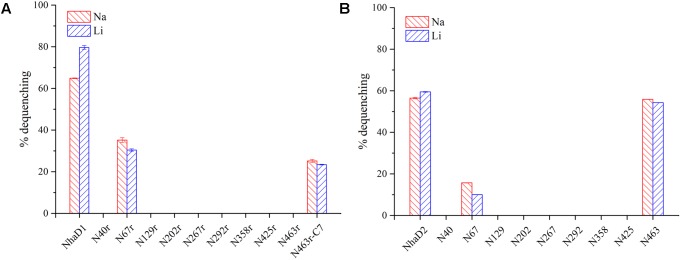
Fluorescence-based antiport activities of chimerical proteins in sub-bacterial vesicles. **(A)** Na^+^ and Li^+^ antiport activities of NhaD1 chimerical proteins. **(B)** Na^+^ and Li^+^ antiport activities of NhaD2 chimerical proteins. The transport activities were measured in the assay buffer containing 10 mM Tris-MES (pH 8.5), 140 mM choline chloride, 5 mM MgCl_2_, 1 μl acridine orange and 60 μg of vesicle protein. The antiporter activity was measured from the dequenching of fluorescence upon the subsequent addition of 10 mM of Na^+^, or Li^+^.

**FIGURE 3:**
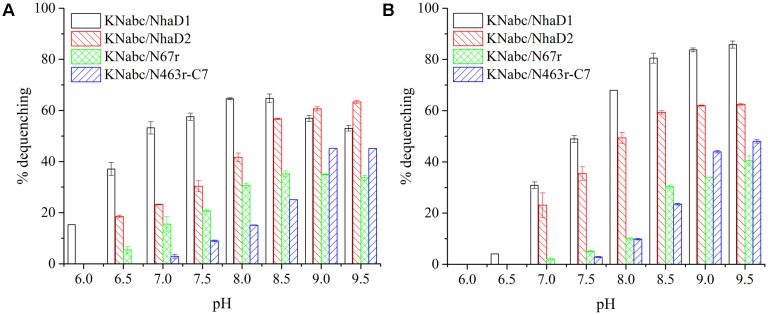
Fluorescence-based antiport activities of NhaD1 chimerical proteins in sub-bacterial vesicles. **(A)** Transport activities of NhaD1 chimeric proteins with a supplementation of 10 mM NaCl at various pHs. **(B)** Transport activities of NhaD1 chimeric proteins with a supplementation of 10 mM LiCl at various pHs.

The generated chimerical strains are shown in **Table 1**. To test their complementation abilities, growth was investigated in Luria–Bertani medium containing various concentrations of 200 or 300 mM NaCl. Similarly, all strains carrying NhaD1 chimeras, except KNabc/N67r, displayed identical ion resistance to that of KNabc/NhaD1 (**Table 2**). Moreover, these fusion proteins abolished transport activity in an everted membrane assay (**Figure 2**). In contrast, the KNabc/N67r fusion strain could tolerate up to 300 mM Na^+^ and the protein N67r retained modest activities for Na^+^ and Li^+^ transport, with a similar pH profile to that of NhaD1 and maximum activity at pH 8.5–9.0 (**Figure 3**).

**Table 1:** Strains used or generated in this study.

Strain	Relevant phenotype or genotype	Reference
*Halomonas* sp. Y2	Moderate halophilic alkalophilic strain	Yang et al., 2010
*E. coli* C43	Competent cell for expression	
*E. coli* DH5α	Competent cell for cloning	
*E. coli* KNabc	Δ*nhaA*, Δ*nhaB*, and Δ*chaA*	Nozaki et al., 1998
**NhaD1 chimerical strain**		
KNabc/N40r	*E. coli* KNabc with N40r	This study
KNabc/N67r	*E. coli* KNabc with N67r	This study
KNabc/N129r	*E. coli* KNabc with N129r	This study
KNabc/N202r	*E. coli* KNabc with N202r	This study
KNabc/N267r	*E. coli* KNabc with N267r	This study
KNabc/N292r	*E. coli* KNabc with N292r	This study
KNabc/N358r	*E. coli* KNabc with N358r	This study
KNabc/N425r	*E. coli* KNabc with N425r	This study
KNabc/N463r	*E. coli* KNabc with N463r	This study
**NhaD2 chimerical strain**		
KNabc/N40	*E. coli* KNabc with N40	This study
KNabc/N67	*E. coli* KNabc with N67	This study
KNabc/N129	*E. coli* KNabc with N129	This study
KNabc/N202	*E. coli* KNabc with N202	This study
KNabc/N267	*E. coli* KNabc with N267	This study
KNabc/N292	*E. coli* KNabc with N292	This study
KNabc/N358	*E. coli* KNabc with N358	This study
KNabc/N425	*E. coli* KNabc with N425	This study
KNabc/N463	*E. coli* KNabc with N463	This study
KNabc/N463r-C7	*E. coli* KNabc with N463r-C7	This study
**N463r-C7 mutant**		
KNabc/N463r-C7-V466A	*E. coli* KNabc with N463r-C7-V466A	This study
KNabc/N463r-C7-A468V	*E. coli* KNabc with N463r-C7-A468V	This study
KNabc/N463r-C7-V474I	*E. coli* KNabc with N463r-C7-V474I	This study
KNabc/N463r-C7-A478I	*E. coli* KNabc with N463r-C7-A478I	This study
KNabc/N463r-C7-A479V	*E. coli* KNabc with N463r-C7-A479V	This study
KNabc/N463r-C7-M482W	*E. coli* KNabc with N463r-C7-M482W	This study
KNabc/N463r-C7-I483L	*E. coli* KNabc with N463r-C7-I483L	This study
***Halomonas* sp. Y2 strain**		
Y2/Δ*nhaD2*	Y2 with *nhaD2* deficiency	Cheng et al., 2016
Y2/NhaD2	Y2/Δ*nhaD2* with NhaD2	Cheng et al., 2016
Y2/N463r-C7	Y2/Δ*nhaD2* with N463r-C7	This study
Y2/N463r-C7-V466A	Y2/Δ*nhaD2* with N463r-C7-V466A	This study
Y2/N463r-C7-A468V	Y2/Δ*nhaD2* with N463r-C7-A468V	This study
Y2/N463r-C7-V474I	Y2/Δ*nhaD2* with N463r-C7-V474I	This study
Y2/N463r-C7-A478I	Y2/Δ*nhaD2* with N463r-C7-A478I	This study
Y2/N463r-C7-A479V	Y2/Δ*nhaD2* with N463r-C7-A479V	This study
Y2/N463r-C7-M482W	Y2/Δ*nhaD2* with N463r-C7-M482W	This study
Y2/N463r-C7-I483L	Y2/Δ*nhaD2* with N463r-C7-I483L	This study
***E. coli* C43 strains FRET analysis**		
C43/D2-CFP-YFP	*E. coli* C43 with D2-CFP-YFP	Meng et al., 2017
C43/N463r-CFP-YFP	*E. coli* C43 with N463r-CFP-YFP	This study

**Table 2:** Growth of constructed *E. coli* KNabc chimeras in the LBK medium.

Strain	NaCl (mM)		Strain	NaCl (mM)	
	200	300		200	300
KNabc/NhaD1	0.86 ± 0.05	0.05 ± 0.01	KNabc/NhaD2	1.62 ± 0.10	1.21 ± 0.11
KNabc/N40r	0.35 ± 0.02	0.07 ± 0.01	KNabc/N40	0.33 ± 0.01	0.06 ± 0.01
KNabc/N67r	1.20 ± 0.09	0.25 ± 0.02	KNabc/N67	0.83 ± 0.06	0.25 ± 0.04
KNabc/N129r	0.81 ± 0.06	0.07 ± 0.02	KNabc/N129	0.98 ± 0.09	0.05 ± 0.01
KNabc/N202r	0.73 ± 0.02	0.05 ± 0.01	KNabc/N202	0.83 ± 0.04	0.06 ± 0.01
KNabc/N267r	0.97 ± 0.03	0.06 ± 0	KNabc/N267	0.67 ± 0.07	0.06 ± 0.01
KNabc/N292r	0.58 ± 0.02	0.05 ± 0.01	KNabc/N292	0.89 ± 0.04	0.06 ± 0.01
KNabc/N358r	0.56 ± 0.04	0.04 ± 0	KNabc/N358	0.92 ± 0.05	0.19 ± 0.03
KNabc/N425r	0.74 ± 0.03	0.08 ± 0.01	KNabc/N425	0.80 ± 0.06	0.22 ± 0.01
KNabc/N463r	0.49 ± 0.05	0.03 ± 0.01	KNabc/N463	0.82 ± 0.03	0.06 ± 0.01
KNabc/N463r-C7	0.78 ± 0.02	0.19 ± 0.01	KNabc/pEASY	0.06 ± 0.01	0.05 ± 0.01

*The medium contains indicated concentrations of NaCl or LiCl and measured at 600 nm (OD_600_) after 24 h of cultivation. Each strain was tested in three replicates.*

**FIGURE 4:**
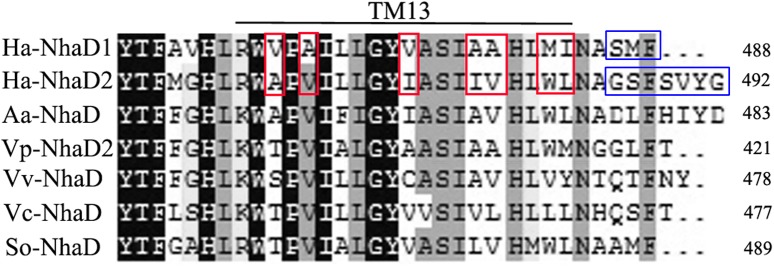
Multiple alignment of the amino terminal sequences of various NhaD homologs by Clustal_X. Residues in blue squares are the replaced residues for chimera N463r-C7 construction; residues in TM XIII for mutation are shown in red squares. Amino acid sequence of NhaD2 was used as a query sequence for BlastP analysis. Homologous sequences from *Alkalimonas amylolytica* (Aa-NhaD, No. Q56EB3), *Vibrio parahaemolyticus* (Vp-NhaD), *Vibrio vulnificus* (Vv-NhaD, No. OJI52092), *Vibrio cholera* (Vc-NhaD, No. A5F120), and *Shewanella oneidensis* (So-NhaD, No. Q8EIA8) were selected for alignments.

The substitution of residues 1–463 in NhaD1 with the corresponding region from NhaD2 (chimeric N463r, **Figure 1**) completely abolished the transport activity and complementation ability, indicating the importance of the C-terminal region of NhaD antiporters (**Figure 2**). To recover the complementation ability of N463r, the three C-terminal residues of N463r (SMF) were replaced with seven C-terminal residues of NhaD2 (GSFSVYG) (shown in the blue squares of **Figure 4**), and a fusion protein of N463r-C7 was generated (**Figure 1**). A weak ion resistance was obtained for the chimeric KNabc/N463r-C7 strain, with a slight growth observed (OD_600_ = 0.19) upon 300 mM Na^+^ stress (**Table 2**). In contrast to the weak restored complementary growth, highly restored activities for Na^+^ and Li^+^ transport were detected in the everted membrane vesicle, with around 50% dequenching activities observed at pH 9.5. In addition, N463r-C7 exhibited a similar pH profile to that of NhaD2, in which the highest activity for Na^+^(Li^+^) transport was observed at pH 9.5 or higher (**Figure 3**).

### *E. coli* KNabc Chimeras of NhaD2

**FIGURE 5:**
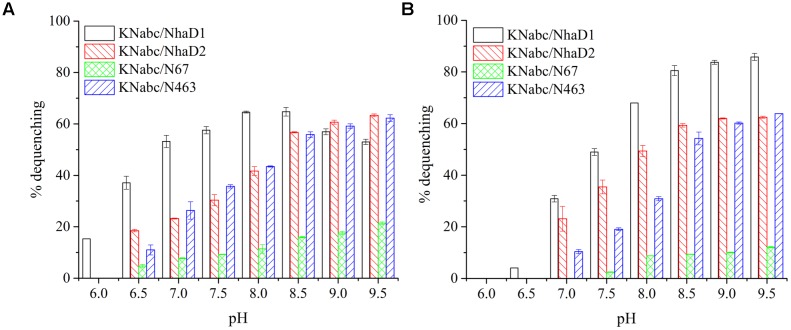
Fluorescence-based antiport activities of NhaD2 chimerical proteins in sub-bacterial vesicles. **(A)** Transport activities of NhaD2 chimeric proteins with a supplementation of 10 mM NaCl at various pHs. **(B)** Transport activities of NhaD2 chimeric proteins with a supplementation of 10 mM LiCl at various pHs.

Compared to wild-type NhaD2, most chimeras displayed a remarkable reduction in growth complementation, with no growth detected in the presence of 300 mM NaCl. Differently, the *E. coli* KNabc strains carrying fusions of N67, N358, and N425 showed weak resistance to 300 mM NaCl (**Table 2**). In agreement with their impaired complementation, most fusion proteins abolished dequenching activities in the everted membrane assay, with only two exceptions, i.e., N67 and N463 chimeric proteins (**Figure 2**). Consistent with the weak growth complementation, modest Na^+^ and Li^+^ activities were detected in the everted membrane of N67, which exhibits a very similar pH-dependence to that of NhaD2. Interestingly, another active chimera of N463 retained full transport activity as well as a similar pH profile to that of NhaD2 (**Figures 5A,B**), albeit of its weak ion resistant abilities. Taken together, we proposed that the C-terminal region functions in both transport activity and the pH response of NhaD2.

### Mutations in the TM XIII Region

As shown in **Figure 1**, the generated chimeric fragment of N463r-C7 comprises almost the full length of NhaD2 antiporter, except for the substitution of residues 463–485 with the corresponding region of NhaD1. Its weak complementation suggested that region 463–485 is indispensable for the robust complementation of NhaD2. Therefore, we compared this region in NhaD1 and NhaD2 to identify non-conserved residues for targeted site-directed mutagenesis (**Figure 4**). Using N463r-C7 as the template, seven residues were successively replaced with the corresponding residues of NhaD2, and their *E. coli* KNabc transformants were further evaluated for complementation ability and transport activity.

**FIGURE 6:**
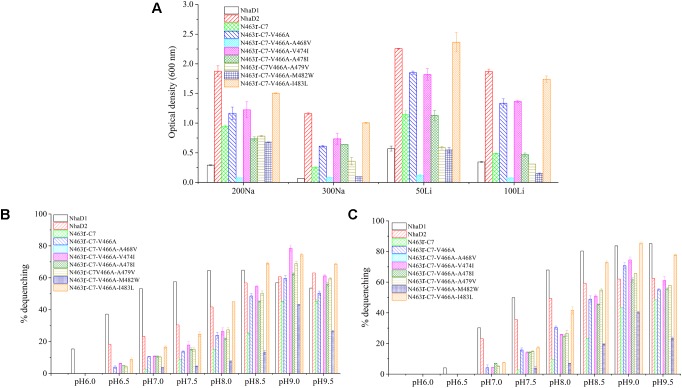
Growth and antiport activities of N463r-C7 mutants in TM XIII. **(A)** Growth of mutants in the LBK medium containing indicated concentrations of NaCl or LiCl. **(B)** Fluorescence-based assays of mutants which initiated by 10 mM Na^+^ and measured at different pH values. **(C)** Fluorescence-based assays of mutants which initiated by 10 mM Li^+^ and measured at different pH values.

As expected, the last mutant KNabc/N463r-C7-I483L, which carried the NhaD2 sequence, exhibited identical complementation ability and dequenching activity to those of NhaD2. In addition, other than the A468V and M482W variants, all other mutants displayed obviously higher resistance to Na^+^ and Li^+^ ions than that of NhaD1 (**Figure 6A**). As shown in **Figures 6B,C**, NhaD1 exhibited high Na^+^-expelling activity in acidic pH conditions, whereas other variants and NhaD2 were active at pH 9.0–9.5. In the vesicle assays, these seven variants showed an intermediate-type pH response between those of NhaD1 and NhaD2, but is closely related with that of NhaD2.

**FIGURE 7:**
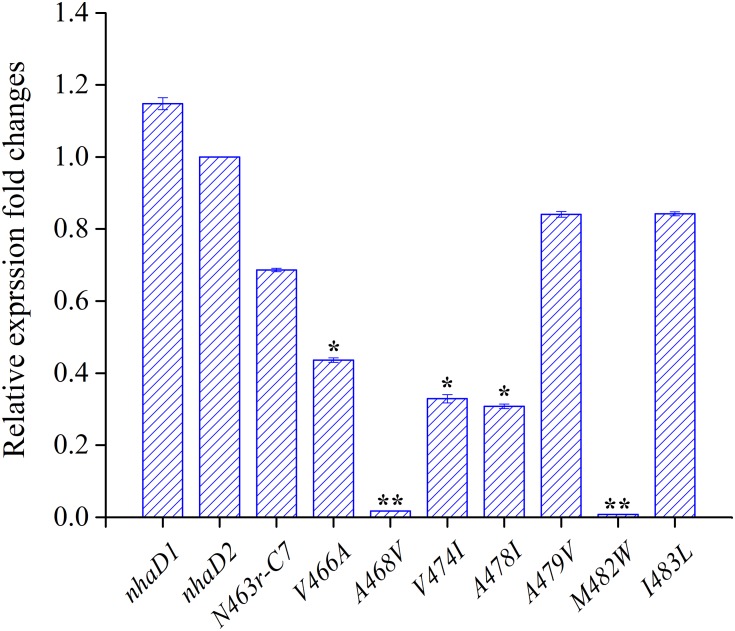
Transcript levels of N463r-C7 mutants determined by qPCR analysis. The relative expression levels for each gene were calculated using the by 2^-ΔΔCt^ with normalization to *16S rRNA* genes. The significant differences (^∗∗^*P* < 0.001, ^∗^*P* < 0.05, fold change ≥2 or fold change ≤0.5, Student’s *t*-test) between the expression levels of mutated genes and *nhaD2* are indicated by asterisks.

During the construction of seven variants, the recovery of growth complementation did not display an upward trend, as expected, from KNabc/N463r-C7 to KNabc/N463r-C7-I483L. Indeed, most of chimerical strains showed an obviously enhanced ion resistance than that of KNabc/N463r-C7, and the last mutant KNabc/N463r-C7-I483L restored the robust complementary ability of NhaD2. However, a remarkable exception at site 468 (A468V) was noticeable, which failed to complement the ion resistance of *E. coli* KNabc and completely abolished transport activity, although we constructed several chimeric clones for confirmation. Additionally, the mutant KNabc/N463r-C7-M482W possessed a relative weak resistance to both Na^+^ and Li^+^ (OD_600_ = 0.095 in the presence of 300 mM NaCl), as well as lower dequenching activities (13.0% and 19.5% for Na^+^ and Li^+^, pH 8.5). For explanation, qPCR was used to monitor the expression levels of these mutants, by using the expression level of *nhaD2* as a baseline. As a result, the expression levels of *nhaD1* and *nhaD2* are identical. Remarkably, the transcript levels of A468V and M482W showed a significant downregulation than those of other targeted genes (**Figure 7**), indicating that poor transport activities of these two mutants should be attributed to their decreased transcript levels in the KNabc strain.

To further assess the affinity of seven variants to alkali cations, the apparent *K*_m_ values were determined in their sub-bacterial vesicles (**Table 3**). The apparent *K*_m_ value of M482W for Na^+^ and Li^+^ binding was two to three times higher than that of wild-type NhaD2. The reduced binding affinity may also partially explain the decreased complementation of *E. coli* KNabc/M482W for Na^+^ and Li^+^ resistance. All other active mutants exhibited similar *K*_m_ values to that of NhaD2, indicating that ion binding was not affected by these site mutations.

**Table 3:** Apparent *K_m_* values of constructed fusion proteins in TM XIII.

Variants	Apparent *K_m_* (mM)	
	Na^+^	Li^+^
NhaD1	0.89 ± 0.05	2.43 ± 0.12
NhaD2	0.47 ± 0.02	2.07 ± 0.11
N463r-C7	0.33 ± 0.01	1.44 ± 0.12
N463r-C7-V466A	0.49 ± 0.05	2.29 ± 0.08
N463r-C7-A468V	–	–
N463r-C7-V474I	0.48 ± 0.02	2.95 ± 0.06
N463r-C7-A478I	0.42 ± 0.03	2.82 ± 0.11
N463r-C7-A479V	0.37 ± 0.02	2.64 ± 0.14
N463r-C7-M482W	1.67 ± 0.06	5.00 ± 0.21
N463r-C7-I483L	0.59 ± 0.05	2.48 ± 0.08

*The everted membrane activities of these proteins were measured in buffer B solution at pH 8.5, with different concentrations of NaCl or LiCl (0.01–100 mM) supplementation. “–” indicates no activity and not determined. The activity of each sample was determined in triplicate.*

### Ion Resistance of Complementary Strains of *Halomonas* sp. Y2/*ΔnhaD2*

To verify the physiological functions of TM XIII in strain *Halomonas* sp. Y2, the seven mutated plasmids of N463r-C7 were transformed into the NhaD2-deficient strain we previously constructed (Cheng et al., 2016, designated Y2/*ΔnhaD2* in the text) and generated seven complementary strains (**Table 1**). For comparison, N463r-C7 was also inserted into Y2/*ΔnhaD2* and cultured under the same conditions. As shown in **Table 4**, the growth of the NhaD2-deficient strain was seriously inhibited by 15% NaCl at both tested pHs, whereas partial or completely restored growth was detected in the complementary strain Y2/*nhaD2*. In compared to NhaD2, the *nhaD1*-disrupted strain was merely modestly sensitive to 15% NaCl at pH 10.0. These results are consistent with our previous conclusions that NhaD2 plays important roles in the regulation of ion homeostasis (Cheng et al., 2016). Under high pH and ion concentrations, all complement strains except Y2/N463r-C7-A468V exhibited obviously greater growth than that of Y2/N463r-C7. Moreover, identical growth to that of wild-type *Halomonas* sp. Y2 was detected in the final strain (Y2/N463r-C7-I483L), with a similar OD_600_ values at pH 8.0 (OD_600_ = 2.17) and pH 10.0 (OD_600_ = 1.08), in the presence of 15% NaCl (**Table 4**). These *in situ* capacities for ion resistance are in good agreement with those of *E. coli* KNabc recombinants (**Figure 6A**), including the impaired ion resistance of A468V mutants. It is noticeable that the impaired growth was mainly observed in the highly alkaline and saline medium (15% NaCl, pH 10.0), in which Y2/N463r-C7-A468V showed a high similar growth to that of Y2/*ΔnhaD2*.

**Table 4:** Complementation growth of constructed mutants to the wild type *Halomonas* sp. Y2.

Strain	pH 8.0			pH 10.0	
	10% Na	15% Na	6% Li	10% Na	15% Na
*Halomonas* sp. Y2	5.71 ± 0.31	2.18 ± 0.07	0.91 ± 0.12	4.87 ± 0.19	1.23 ± 0.08
Y2/Δ*nhaD1*	5.57 ± 0.12	2.29 ± 0.02	0.89 ± 0.05	4.46 ± 0.11	0.65 ± 0.07
Y2/Δ*nhaD2*	4.26 ± 0.21	1.09 ± 0.02	0.78 ± 0.05	3.32 ± 0.12	0.24 ± 0.04
Y2/Δ*nhaD1*/*nhaD1*	5.01 ± 0.23	2.05 ± 0.10	0.95 ± 0.03	4.69 ± 0.17	1.02 ± 0.06
Y2/Δ*nhaD2*/*nhaD2*	5.59 ± 0.19	1.76 ± 0.11	0.90 ± 0.12	4.90 ± 0.15	0.93 ± 0.08
Y2/N463r-C7	4.01 ± 0.14	0.66 ± 0.03	0.42 ± 0.04	4.50 ± 0.17	0.14 ± 0.01
Y2/N463r-C7-V466A	4.59 ± 0.17	1.23 ± 0.12	0.34 ± 0.02	4.06 ± 0.09	0.29 ± 0.02
Y2/N463r-C7-A468V	4.98 ± 0.20	1.41 ± 0.09	0.36 ± 0.06	4.35 ± 0.04	0.20 ± 0.02
Y2/N463r-C7-V474I	4.30 ± 0.21	1.47 ± 0.10	0.68 ± 0.11	4.18 ± 0.20	0.54 ± 0.03
Y2/N463r-C7-A478I	4.47 ± 0.14	1.54 ± 0.12	0.69 ± 0.08	4.41 ± 0.15	0.41 ± 0.06
Y2/N463r-C7-A479V	4.90 ± 0.17	1.50 ± 0.08	0.48 ± 0.07	4.38 ± 0.12	0.58 ± 0.09
Y2/N463r-C7-M482W	4.44 ± 0.16	1.65 ± 0.18	0.82 ± 0.12	3.38 ± 0.18	0.51 ± 0.07
Y2/N463r-C7-I483L	5.10 ± 0.22	2.17 ± 0.21	0.84 ± 0.08	4.49 ± 0.21	1.08 ± 0.12

*The mutated fragments were from plasmid N463r-C7 and transformed into strain Halomonas sp. Y2 with NhaD2 deficiency (Y2/ΔnhaD2). After 24-h incubation in the LB medium with various concentrations of NaCl and LiCl, OD_600nm_ were determined. Each strain was tested in three replicates.*

### FRET Analysis for Conformational Changes of N463r Fusion

**FIGURE 8:**
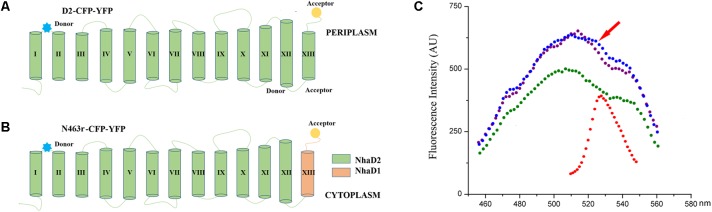
Schematic representation and fluorescence spectra of cell suspensions containing N39 fusions for FRET analysis. **(A)** NhaD2 fusion protein with CFP fused at site 39 and YFP at the C terminus. **(B)** N463r fusion protein with CFP fused at site 39 and YFP at the C terminus. **(C)** Fluorescence spectra of CFP and YFP constructs. Fluorescence emissions upon excitation by 433 nm for CFP or 473 nm for YFP were measured in the cells that resuspending in Tricine-KOH buffer (pH 8.5). Fluorescence emission upon 473 nm excitation of D2-CFP-YFP was recorded as red; fluorescence emission of D2-CFP fusion cells that excited by 433 nm was recorded as green; fluorescence emission upon 433 nm excitation of D2-CFP-YFP was presented as blue; and fluorescence emission upon 433 nm excitation of N463r-CFP-YFP was presented as purple.

It is well accepted that FRET could be used to monitor protein–protein interactions, oligomerization, and conformational change of proteins (Stryer, 1978; Karasawa et al., 2005). Based on a previous FRET construct of N39-CFP-C-YFP (designed D2-CFP-YFP in this study, **Figure 8A**) as a template (Meng et al., 2017), we successfully replaced its C-terminal region (residues 464–492) with the corresponding fragment of NhaD1 and generated chimeric protein N463r-CFP-YFP (**Figures 8A,B**). After cultivation and induction, the suspended cells were subjected to the fluorescence scanning from 400 to 600 nm. Compared to D2-CFP-YFP, the fluorescence spectrum of N463r-CFP-YFP displayed a reduced signal at 528 nm (red arrow) when being excitated by 433 nm. This was identical to the negative control D2-N39-CFP, but much lower than that of D2-CFP-YFP (**Figure 8C**). These results suggest that the substitution of region 464–492 induced some conformational changes and prolonged the distance between TM I and C-terminus.

## Discussion

In this study, taking advantage of the high sequence identity of NhaD1 and NhaD2, various chimeric NhaD1 and NhaD2 fusions were constructed and alterations in transport activity were evaluated. As the major difference between NhaD1 and NhaD2 is their complementation ability, the growth of each *E. coli* KNabc chimera was first tested using different NaCl concentrations. Unexpectedly, most chimeras showed weak growth in the presence of 200 mM NaCl and completely lost their original activities. However, an interesting exception was found in the NhaD2-derived chimera N67, which contains 67 N-terminal residues of NhaD1 and the 68–492 fragment of NhaD2. When compared to NhaD2, it retained partial complementation ability and antiport activity, as well as a similar pH-dependent profile for Na^+^ and Li^+^ translocation. Its corresponding chimera N67r exhibited a slightly higher complementation ability than that of NhaD1, together with partial antiport activity and a similar pH profile and ion affinities to those of NhaD1. In combination with the lack of growth and activities observed for N40 and N40r, we speculate that (i) TMs I and II in the N-terminus functional interact to retain the active structure of NhaD; (ii) region 1–67 is partially responsible for the complementation ability of the antiporter, but is not directly involved in the catalytic center for pH sensitivity or ion binding; (iii) region 68–488 in NhaD1 (or 492 in NhaD2) is the catalytic center for pH sensitivity and ion translocation.

Another remarkable exception is two fusions at site 463, in which N463r completely abolished the transport activity and complementation abilities. We previously disclosed that the C-terminus of NhaD1 and NhaD2 is irreplaceable for their ion transport capacities (Meng et al., 2017), and consequently, the diminished activity of N463r was expected. In contrast, N463 retained a surprising high activity and similar pH profile as that of NhaD2. As shown in **Figure 1**, chimera N463 comprises almost the full length of NhaD1, except for the substitution of region 464–488 with the corresponding region (464–492) of NhaD2. Therefore, we suspected that the C-terminal region is directly involved in the pH sensitivity of these antiporters and structurally affects the transport activity. Contrast to robust dequenching activity, the KNabc strain carrying chimera N463 plasmid only displayed slightly complementation ability. Such gaps between the transport activity and ion complementary ability is similar to that of wild-type NhaD1. Possible explanation for such gaps would be the difference of *in vivo* and *in vitro* environments, i.e., the Tris-MES assay mixture is different from the physiological environment of the cells. In the pH-blocked structure of Ec-NhaA, the periplasmic passage is blocked by an ion barrier under some conditions (Karpel et al., 1988; Hunte et al., 2005). Thereby, different environments might affect the pH-activated conformational changes of these proteins and retard the ion translocation process. In combination with the alterations of pH profiles of those chimeras, we suspected that region 464–492 might be critical to the pH sensitivity and pH activation of NhaD2 antiporter.

To explore critical regions that are responsible for the strong physiological functions of NhaD2, N463r-C7 was further generated by replacing its four C-terminal amino acids with seven amino acids from NhaD2. Notably, the transport activity was significantly enhanced by this fusion; around 50% activity was observed at pH 9.5 in the everted membrane of N463r-C7 (**Figure 3**). These recoveries agree well with our previous conclusion that the C-terminus of NhaD functionally interacts with the N-terminus (Meng et al., 2017). However, strains of *E. coli* KNabc and *Halomonas* sp. Y2/*ΔnhaD2* carrying N463r-C7 merely displayed a weakly restored ion resistance (**Tables 2**, **4**). As shown in **Figure 1**, the majority of the N463r-C7 fragment was from NhaD2, whereas only residues 464–485 were replaced with the corresponding sequence in NhaD1. Therefore, the weakly recovered complementation ability implies that region 464–485 is important to the robust *in vivo* functions of NhaD2.

Based on the constructed topological model of NhaD2 (Meng et al., 2017), residues 464–485 are mainly located in the last helix of TM XIII, which raises the possibility that TM XIII is a key region for the physiological functions of NhaD2. Seven mutated N463r-C7 plasmids were then constructed and transformed into *E. coli* KNabc or *Halomonas* sp. Y2/aaa*nhaD2*. Compared to KNabc/N463r-C7 and Y2/aaa*nhaD2*/N463r-C7, the ion resistances of these mutants were significantly improved. Considering the seriously impaired FRET efficiency of N463r-CFP-YFP, we proposed that the substitution of region 464–482 caused a conformational change in NhaD2 and seriously blocked its ion translocation function. Further mutations in TM XIII verified our supposition, in which seven mutants recovered the robust transport activities of NhaD2 gradually. Since the pioneering work resolving the crystal structure of Ec-NhaA, structures of other bacterial ion-coupled transporters have been determined (Hunte et al., 2005). These include some ATPases (Morth et al., 2007; Olesen et al., 2007), secondary transporters of Na^+^/galactose (Faham et al., 2008), Na^+^/bile acid (Hu et al., 2011), and Na^+^/citrate (Mancusso et al., 2014). These antiporters share little or no similarity to Ec-NhaA, but also include inverted topological repeats containing an interrupted helix where the ion-binding site is located (Screpanti and Hunte, 2007). A series of charged/polar mutations in Vc-NhaD also suggested that NhaD antiporters may share common structural features and catalytic modes with other reports (Habibian et al., 2005). Based on these resolved structures, the last helix is not directly involved in ion translocation. Similar to those of Ec-NhaA, TMs IV, V, VI, and X-XII in Vc-NhaD are suggested to form a transmembrane relay involved in the attraction, coordination, and translocation of transported cations (Habibian et al., 2005). We speculated that NhaD1 and NhaD2 contain a similar relay as that of NhaA, and TM XIII is not directly involved in the catalytic cavity but conformational affects the ion barrier and pH activation of NhaD antiporters. In the physiological environments, the conformational activations of NhaD1 and N463 were affected and thereby retards the *in vivo* transport ability.

## Conclusion

Taken together, high *in vitro* activities of NhaD1 and NhaD2 and the remarkable difference of *in vivo* ion resistance provided an interesting starting point for studying the transport mechanism of NhaD-type antiporters. It is likely that the mechanism underlying the robust physiological function of NhaD2 includes multiple components, but the N and C terminal regions, especially TM XIII, is indispensable. We suspected that TM XIII is in close proximity to the periplasmic catalytic center of NhaD2, and consequently influences the conformational change of the pH-activated or ion-translocating states that observed in Ec-NhaA or MjNhaP_1_ (Olkhova et al., 2006; Schushan et al., 2012; Cristina and Werner, 2014).

## Author Contributions

CY designed the experiments and analyzed the data. CY and PX prepared the manuscript. ZY, YM, QZ, and BC conducted the experiments.

## Conflict of Interest Statement

The authors declare that the research was conducted in the absence of any commercial or financial relationships that could be construed as a potential conflict of interest.

## Footnotes

1 http://www.tcdb.org

## References

[B1] Appel M. Hizlan D. Vinothkumar K. R. Ziegler C. Kühlbrandt W. (2009). Conformations of NhaA, the Na^+^/H^+^ exchanger from *Escherichia coli*, in the pH-activated and ion-translocating states. *J. Mol. Biol.* 386 351–365. 10.1016/j.jmb.2008.12.042 19135453

[B2] Cheng B. Meng Y. W. Cui Y. B. Li C. F. Tao F. Yin H. J. (2016). Alkaline response of a halotolerant alkaliphilic *Halomonas* strain and functional diversity of its Na^+^(K^+^)/H^+^ antiporters. *J. Biol. Chem.* 291 26056–26065. 10.1074/jbc.M116.751016 27777302 PMC5207076

[B3] Cristina P. Werner K. (2014). pH- and sodium-induced changes in a sodium/proton antiporter. *eLife* 3:e01412. 10.7554/eLife.01412 24473071 PMC3900740

[B4] Cui Y. B. Cheng B. Meng Y. W. Li C. F. Yin H. J. Xu P. (2016). Expression and functional analysis of two NhaD type antiporters from the halotolerant and alkaliphilic *Halomonas* sp. Y2. *Extremophiles* 20 631–639. 10.1007/s00792-016-0852-8 27315164

[B5] Dzioba J. Ostroumov E. Winogrodzki A. Dibrov P. (2002). Cloning, functional expression in *Escherichia coli* and primary characterization of a new Na^+^/H^+^ antiporter, NhaD, of *Vibrio cholerae*. *Mol. Cell. Biochem.* 229 119–124. 10.1023/A:1017932829927 11936836

[B6] Faham S. Watanabe A. Besserer G. M. Cascio D. Specht A. Hirayama B. A. (2008). The crystal structure of a sodium galactose transporter reveals mechanistic insights into Na^+^/sugar symport. *Science* 321 810–814. 10.1126/science.1160406 18599740 PMC3654663

[B7] Goldberg E. B. Arbel T. Chen J. Karpel R. Mackie G. A. Schuldiner S. (1987). Characterization of a Na^+^/H^+^ antiporter gene of *Escherichia coli*. *Proc. Natl. Acad. Sci. U.S.A.* 84 2615–2619. 10.1073/pnas.84.9.2615 3033655 PMC304708

[B8] Habibian R. Dzioba J. Barrett J. Galperin M. Y. Loewen P. C. Dibrov P. (2005). Functional analysis of conserved polar residues in Vc-NhaD, Na^+^/H^+^ antiporter of *Vibrio cholerae*. *J. Biol. Chem.* 280 39637–39643. 10.1074/jbc.M509328200 16186100

[B9] Herz K. Rimon A. Olkhova E. Kozachkov L. Padan E. (2010). Transmembrane segment II of NhaA Na^+^/H^+^ antiporter lines the cation passage, and Asp65 is critical for pH activation of the antiporter. *J. Biol. Chem.* 285 2211–2220. 10.1074/jbc.M109.047134 19923224 PMC2804377

[B10] Hu N. J. Iwata S. Cameron A. D. Drew D. (2011). Crystal structure of a bacterial homologue of the bile acid sodium symporter ASBT. *Nature* 478 408–411. 10.1038/nature10450 21976025 PMC3198845

[B11] Hunte C. Screpanti M. Venturi M. Rimon A. Padan E. Michel H. (2005). Structure of a Na^+^/H^+^ antiporter and insights into mechanism of action and regulation by pH. *Nature* 534 1197–1202. 10.1038/nature03692 15988517

[B12] Karasawa A. Tsuboi Y. Inoue H. Kinoshita R. Nakamura N. Kanazawa H. (2005). Detection of oligomerization and conformational changes in the Na^+^/H^+^ antiporter from *Helicobacter pylori* by fluorescence resonance energy transfer. *J. Biol. Chem.* 280 41900–41911. 10.1074/jbc.M510795200 16216867

[B13] Karpel R. Olami Y. Taglicht D. Schuldiner S. Padan E. (1988). Sequencing of the gene ant which affects the Na^+^/H^+^ antiporter activity in *Escherichia coli*. *J. Biol. Chem.* 263 10408–10414. 2839489

[B14] Kozachkov L. Herz K. Padan E. (2007). Functional and structural interactions of the transmembrane domain X of NhaA, Na^+^/H^+^ antiporter of *Escherichia coli*, at physiological pH. *Biochemistry* 46 2419–2430. 10.1021/bi602393s 17284054

[B15] Krulwich T. A. Sachs G. Padan E. (2011). Molecular aspects of bacterial pH sensing and homeostasis. *Nat. Rev. Microbiol.* 9 330–343. 10.1038/nrmicro2549 21464825 PMC3247762

[B16] Kurz M. Brünig A. N. Galinski E. A. (2006). NhaD type sodium/proton-antiporter of *Halomonas elongata*: a salt stress response mechanism in marine habitats? *Saline Systems* 2:10. 10.1186/1746-1448-2-10 16872527 PMC1552076

[B17] Liu J. Xue Y. Wang Q. Wei Y. Swartz T. H. (2005). The activity profile of the NhaD-type Na^+^(Li^+^)/H^+^ antiporter from the soda lake haloalkaliphile *Alkalimonas amylolytica* is adaptive for the extreme environment. *J. Bacteriol.* 187 7589–7595. 10.1128/JB.187.22.7589-7595.2005 16267283 PMC1280297

[B18] Maes M. Rimon A. Kozachkov-Magrisso L. Friedler A. Padan E. (2012). Revealing the ligand binding site of NhaA Na^+^/H^+^ antiporter and its pH dependence. *J. Biol. Chem.* 287 38150–38157. 10.1074/jbc.M112.391128 22915592 PMC3488084

[B19] Mager T. Rimon A. Padan E. Fendler K. (2011). Transport mechanism and pH regulation of the Na^+^/H^+^ antiporter NhaA from *Escherichia coli*: an electrophysiological study. *J. Biol. Chem.* 286 23570–23581. 10.1074/jbc.M111.230235 21566125 PMC3123120

[B20] Mancusso R. Gregorio G. G. Liu Q. Wang D. N. (2014). Structure and mechanism of a bacterial sodium-dependent dicarboxylate transporter. *Nature* 491 622–626. 10.1038/nature11542 23086149 PMC3617922

[B21] Meng Y. Yang Z. Cheng B. Nie X. Y. Li S. N. Yin H. J. (2017). Functional interaction between the N- and C-termini of NhaD antiporters from *Halomonas* sp. Y2. *J. Bacteriol.* 199 1–11. 10.1128/JB.00302-17 28559301 PMC5527383

[B22] Morth J. P. Pedersen B. P. Toustrup-Jensen M. S. Sorensen T. L. Petersen J. Andersen J. P. (2007). Crystal structure of the sodium-potassium pump. *Nature* 450 1043–1049. 10.1038/nature06419 18075585

[B23] Nozaki K. Kuroda T. Mizushima T. Tsuchiya T. (1998). A new Na^+^/H^+^ antiporter, NhaD, of *Vibrio parahaemolyticus*. *Biochim. Biophys. Acta* 1369 213–220. 10.1016/S0005-2736(97)00223-X 9518619

[B24] Olesen C. Picard M. Winther A. M. Gyrup C. Morth J. P. Oxvig C. (2007). The structural basis of calcium transport by the calcium pump. *Nature* 450 1036–1042. 10.1038/nature06418 18075584

[B25] Olkhova E. Hunte C. Screpanti E. Padan E. Michel H. (2006). Multiconformation continuum electrostatics analysis of the NhaA Na^+^/H^+^ antiporter of *Escherichia coli* with functional implications. *Proc. Natl. Acad. Sci. U.S.A.* 103 2629–2634. 10.1073/pnas.0510914103 16477015 PMC1413810

[B26] Padan E. Landau M. (2016). Sodium-proton (Na^+^/H^+^) antiporters: properties and roles in health and disease. *Met. Ions Life Sci.* 16 391–458. 26860308 10.1007/978-3-319-21756-7_12

[B27] Padan E. Venturi M. Gerchman Y. Dover N. (2001). Na^+^/H^+^ antiporters. *Biochim. Biophys. Acta* 1505 144–157. 10.1016/0304-4173(83)90011-3 11248196

[B28] Schushan M. Rimon A. Haliloglu T. Forrest L. R. Padan E. Ben-Tal N. (2012). A model-structure of a periplasm-facing state of the NhaA antiporter suggests the molecular underpinnings of pH-induced conformational changes. *J. Biol. Chem.* 287 18249–18261. 10.1074/jbc.M111.336446 22431724 PMC3365733

[B29] Screpanti E. Hunte C. (2007). Discontinuous membrane helices in transport proteins and their correlation with function. *J. Struct. Biol.* 159 261–267. 10.1016/j.jsb.2007.01.011 17350860

[B30] Stryer L. (1978). Fluorescence energy transfer as a spectroscopic ruler. *Annu. Rev. Biochem.* 47 819–846. 10.1146/annurev.bi.47.070178.004131 354506

[B31] Thompson J. D. Gibson T. J. Plewniak F. Jeanmougin F. Higgins D. G. (1997). The CLUSTAL_X windows interface: flexible strategies for multiple sequence alignment aided by quality analysis tools. *Nucleic Acids Res.* 25 4876–4882. 10.1093/nar/25.24.4876 9396791 PMC147148

[B32] Tzubery T. Rimon A. Padan E. (2008). Structure based functional study reveals multiple roles of transmembrane segment IX and loop VIII–IX in NhaA Na^+^/H^+^ antiporter of *Escherichia coli* at physiological pH. *J. Biol. Chem.* 283 15975–15987. 10.1074/jbc.M800482200 18387952 PMC3259659

[B33] Yang C. Wang Z. Li Y. Niu Y. Du M. He X. (2010). Metabolic versatility of halotolerant and alkaliphilic strains of *Halomonas* isolated from alkaline black liquor. *Bioresour. Technol.* 101 6778–6784. 10.1016/j.biortech.2010.03.108 20409702

[B34] Zhang H. Wang Z. Wang L. Mu R. Zou Z. Yuan K. (2014). Cloning and identification of a novel NhaD-type Na^+^/H^+^ antiporter from metagenomic DNA of the halophilic bacteria in soil samples around Daban Salt Lake. *Extremophiles* 18 89–98. 10.1007/s00792-013-0600-2 24297704

