# Supplementary material for: Critical Functions of Region 1-67 and Helix XIII in Retaining the Active Structure of NhaD Antiporter in Halomonas sp. Y2
Source: Front Microbiol. 2018 May 2;9:831. doi: 10.3389/fmicb.2018.00831 (PMC5942162; doi:10.3389/fmicb.2018.00831)
Supplement: Supplementary file 1 [file Table_1.DOCX]

**TABLE S1. Plasmids used in this study**

| Plasmid | Relevant phenotype or genotype | Ref. |
| --- | --- | --- |
| pEASYBlunt | Cloning vector | TransGen |
| pET28a | Expression vector | This study |
| pBBR1MCS-5 | Carrier for gene anaplerosis |  |
| NhaD1 | pEASYBlunt with nhaD1 | This study |
| NhaD2 | pEASYBlunt with nhaD2 | This study |
| N40r | pEASYBlunt with chimera N40r | This study |
| N67r | pEASYBlunt with chimera N67r | This study |
| N129r | pEASYBlunt with chimera N129r | This study |
| N202r | pEASYBlunt with chimera N202r | This study |
| N267r | pEASYBlunt with chimera N267r | This study |
| N292r | pEASYBlunt with chimera N292r | This study |
| N358r | pEASYBlunt with chimera N358r | This study |
| N425r | pEASYBlunt with chimera N425r | This study |
| N463r | pEASYBlunt with chimera N463r | This study |
| N40 | pEASYBlunt with chimera N40 | This study |
| N67 | pEASYBlunt with chimera N67 | This study |
| N129 | pEASYBlunt with chimera N129 | This study |
| N202 | pEASYBlunt with chimera N202 | This study |
| N267 | pEASYBlunt with chimera N267 | This study |
| N292 | pEASYBlunt with chimera N292 | This study |
| N358 | pEASYBlunt with chimera N358 | This study |
| N425 | pEASYBlunt with chimera N425 | This study |
| N463 | pEASYBlunt with chimera N463 | This study |
| N463r-C7 | pEASYBlunt with chimera N463r-C7 | This study |
| N463r-C7-V466A | pEASYBlunt with V466A mutant in N463r-C7 | This study |
| N463r-C7-A468V | pEASYBlunt with A468V mutant in N463r-C7 | This study |
| N463r-C7-V474I | pEASYBlunt with V474I mutant in N463r-C7 | This study |
| N463r-C7-A478I | pEASYBlunt with A478I mutant in N463r-C7 | This study |
| N463r-C7-A479V | pEASYBlunt with A479V mutant in N463r-C7 | This study |
| N463r-C7-M482W | pEASYBlunt with M482W mutant in N463r-C7 | This study |
| N463r-C7-I483L | pEASYBlunt with I483L mutant in N463r-C7 | This study |
| D2-CFP | pET28a with *cfp* fused at39 site in *nhaD2* | This study |
| D2-CFP-YFP | pET28a with *cfp* fused at 39 site and *yfp* fused at C-terminus in *nhaD2* | This study |
| N463r-CFP-YFP | pET28a with *cfp* fused at 39 site and *yfp* fused at C-terminus in N463r | This study |
| pECFP-Mito | pECFP-Mito with *cfp* | Clontech |
| pEYFP-N1 | pEYFP-N1 with *yfp* | Clontech |
| pBBR1MCS-5-N463r-C7 | pBBR1MCS-5 with N463r-C7 fusion fragment | This study |
| pBBR1MCS-5-N463r-C7-V466A | pBBR1MCS-5 with N463r-C7-V466A fusion fragment | This study |
| pBBR1MCS-5-N463r-C7-A468V | pBBR1MCS-5 with N463r-C7-A468V fusion fragment | This study |
| pBBR1MCS-5-N463r-C7-V474I | pBBR1MCS-5 with N463r-C7-V474I fusion fragment | This study |
| pBBR1MCS-5-N463r-C7-A478I | pBBR1MCS-5 with N463r-C7-A478I fusion fragment | This study |
| pBBR1MCS-5-N463r-C7-A479V | pBBR1MCS-5 with N463r-C7-A479V fusion fragment | This study |
| pBBR1MCS-5-N463r-C7-M482W | pBBR1MCS-5 with N463r-C7-M482W fusion fragment | This study |
| pBBR1MCS-5-N463r-C7-I483L | pBBR1MCS-5 with N463r-C7-I483L fusion fragment | This study |
