# Supplementary material for: Critical Functions of Region 1-67 and Helix XIII in Retaining the Active Structure of NhaD Antiporter in Halomonas sp. Y2
Source: Front Microbiol. 2018 May 2;9:831. doi: 10.3389/fmicb.2018.00831 (PMC5942162; doi:10.3389/fmicb.2018.00831)
Supplement: Supplementary file 2 [file Table_2.docx]

**TABLE S2. Primers used in this study.** The restriction sites of *Bam*HI, *Hind*III, and *Sal*I were underlined.

| Primer | Sequence |
| --- | --- |
| **NhaD1 chimeras** |  |
| NhaD2 F1 | CGCGGATCCATGTTGACGTTGCATCATGAC |
| NhaD1 R2 | CCCAAGCTTTCAAAACATGCTGGCGTTGA |
| N40r F2 | GGCGAGATCGATCTGACCTCCTCACTG |
| N40r R1 | GGTCAGATCGATCTCGCCCGT |
| N67r F2 | GGCCGAAGAGAAGATCCACATGCGCAAG |
| N67r R1 | CTTGCGCATGTGGATCTTCTCTTCGGCC |
| N129r F2 | CGCCATGGAAGAGCGGCGTGTCTTC |
| N129r R1 | GACACGCCGCTCTTCCATGGCGTTG |
| N202r F2 | TGGCTGCCAATGCAGGGGGCGC |
| N202r R1 | GCGCCCCCTGCATTGGCAGCCAC |
| N267r F2 | TCAAGCGCGGCGCGAAGCGCATC |
| N267r R1 | GCTTCGCGCCGCGCTTGAGCTGCAC |
| N292r F2 | CTGCTGCACCTGCCCCCTGCCATGG |
| N292r R1 | GGCAGGGGGCAGGTGCAGCAGCGTATGG |
| N358r F2 | GTGGGACACACTGCTGTTCTTCTAC |
| N358r R1 | AGAACAGCAGTGTGTCCCACTCGGC |
| N425r F2 | CCACGGGCACTGGCTGCTGATTACC |
| N425r R1 | CAGCAGCCAGTGCCCGTGGGACATGTC |
| N463r F2 | CTTCATGGGGCACCTGCGCTGGG |
| N463r R1 | CCCAGCGCAGGTGCCCCATGAAG |
| **NhaD2 chimeras** |  |
| NhaD1 F1 | CGCGGATCCATGAAACCACCTAACGCCCTCG |
| NhaD2 R2 | CGGGTCGACTCAGCCGTATACCGAAAAGCT |
| N40 F2 | CTGGCCCGCTAGACTTGACCAGTTC |
| N40 R1 | CTGGTCAAGTCTAGCGGGCCAGCAG |
| N67 F2 | GAGTGAAGAAATTATCCACATGCGCAAG |
| N67 R1 | CTTGCGCATGTGGATAATTTCTTCACTC |
| N129 F2 | AACGCCATGGAAGAGCGTCGAGTC |
| N129 R1 | CAAAGACTCGACGCTCTTCCATGGC |
| N202 F2 | GTGGTTGCTTCCAACGCGGGCGGTG |
| N202 R1 | CACCGCCCGCGTTGGAAGCAACCAC |
| N267 F2 | GATGAAGCGCGGTGCACGCCGCATC |
| N267 R1 | GATGCGGCGTGCACCGCGCTTCATC |
| N292 F2 | TCCTGTATCTCCCCCCGGTACTGGGC |
| N292 R1 | AGTACCGGGGGGAGATACAGGATCG |
| N358 F2 | GTGGGACACGCTGCTGTTCTTCTAC |
| N358 R1 | AGAAGAACAGCAGCGTGTCCCACTCGGAGC |
| N425 F2 | GAGCGAAGGCAACTGGCTGTTGATC |
| N425 R1 | CAACAGCCAGTTGCCTTCGCTCATG |
| N463 F2 | CACCTTTGCCGTTCATCTGCGCTGG |
| N463 R1 | CCAGCGCAGATGAACGGCAAAGGTG |
| **NhaD2 mutant** |  |
| 466 R1 | GGCCACATAGCCCAGCAGGATGGCAGGCGCCCAGCGCAGGTGC |
| 466 R2 | CGTTGATCATCAGATGCGCCGCGATACTGGCCACATAGC |
| 466 R3 | GCGTCGACTCAGCCGTATACCGAAAAGCTGCCGGCGTTGATCATC |
| 468 R1 | GGCCACATAGCCCAGCAGGATGACAGGCGCCCAGCGCAGGTGC |
| 474 R1 | AGCCCAGCAGGATGACAGGCGCCCAGCGCAGGTGCC |
| 474 R2 | CAGATGCGCCGCGATACTGGCAATATAGCCCAGCAGG |
| 474 R3 | GCTGCCGGCGTTGATCATCAGATGCGCCGCGATACTG |
| 474 R4 | GCGTCGACTCAGCCGTATACCGAAAAGCTGCCGGCGTTG |
| 478 R1 | GCCCAGCAGGATGACAGGCGCCCAGCGCAGGTGCC |
| 478 R2 | GATCATCAGATGCGCGATGATACTGGCAATATAGCCCAGCAGG |
| 478 R3 | CCGAAAAGCTGCCGGCGTTGATCATCAGATGCG |
| 478 R4 | GCGTCGACTCAGCCGTATACCGAAAAGCTGCCGGCGTTG |
| 479 R2 | GATCATCAGATGCACGATGATACTGGCAATATAGCCCAGCAG |
| 479 R3 | CCGAAAAGCTGCCGGCGTTGATCATCAGATGCACGATGATACTG |
| 482 R1 | GCCCAGCAGGATGACAGGCGCCCAGCGCAGGTGCC |
| 482 R2 | GATGCACGATGATACTGGCAATATAGCCCAGCAGG |
| 482 R3 | CCGAAAAGCTGCCGGCGTTGATCCACAGATGCACGATGATAC |
| 482 R4 | GCGTCGACTCAGCCGTATACCGAAAAGCTGCCGGCGTTG |
| 483 R3 | CCGAAAAGCTGCCGGCGTTGAGCCACAGATGCACGATGATAC |
| NhaD2 R2 | CGGGTCGACTCAGCCGTATACCGAAAAGCT |
| NhaD2 F1 | CGCGGATCCATGTTGACGTTGCATCATGAC |
| **qRT-PCR** |  |
| RT-NhaD1-F | GTCGTCTTGAAGGTGGCTGAG |
| RT-NhaD1-R | GCACCAGCAACGCAAAGAA |
| RT-NhaD2-F | CTAAAGTGGCAGAAGGCGATAA |
| RT-NhaD2-R | TGACCAGCGACGGAATGA |
| RT16S-F | TAATACGGAGGGTGCAAGCG |
| RT16S-R | CTTCGCCACCGGTATTCCTC |
| **FRET analysis** |  |
| NhaD2 F | CGCGGATCCATGTTGACGTTGCATCATGAC |
| YFP F | GAAGTGGAGGAATGGTGAGCAAGGG |
| YFP R | CTTGTCGACTTACTTGTACAGCTCGTCCATG |
| N463r F | CTTCATGGGGCACCTGCGCTGGG |
| N463r R1 | CCCAGCGCAGGTGCCCCATGAAG |
| N463r R2 | TCCTCCACTTCCTCCAAACATGCTGGCGTTGATCA |
| pET28a F | CAAGTAAGTCGACAAGCTTGCGGCCGC |
| pET28a R | CAACGTCAACATGGATCCGCGACCCATTTGCTG |
